# The effect of conflict on child and adolescent health in Amhara region, Ethiopia: Cross-Sectional Study

**DOI:** 10.1186/s12887-023-04282-w

**Published:** 2023-09-14

**Authors:** Gebeyaw Biset, Debrnesh Goshiye, Sisay Gedamu, Mekonnen Tsehay

**Affiliations:** 1 Department of Pediatric and Child Health Nursing, School of Nursing and Midwifery, College of Medicine and Health Sciences, Wollo University, PO. Box 1145, Dessie, Ethiopia; 2 Department of Adult Health Nursing, School of Nursing and Midwifery, College of Medicine and Health Sciences, Wollo University, Dessie, Ethiopia; 3 Department of Psychiatry, College of Medicine and Health Sciences, Wollo University, Dessie, Ethiopia

**Keywords:** Conflict, Trauma/violence, Children and adolescent, Amhara Region, Ethiopia

## Abstract

**Background:**

Currently, conflict become common phenomenon in the world affecting the lives of millions of children. Due the continued conflict in Ethiopia millions of children are suffering from extreme levels of violence, lack of basic humanitarian needs, and lack of health services.

**Objective:**

This study was designed to assess the effect of conflict on child and adolescent health in Amhara region, September 2022.

**Methods:**

A community-based cross-sectional study was employed among children agedd < 18 years in conflict affected areas of Amhara region. The sample size was determined using a single population proportion formula. Data was collected through face-to-face interviews of children or child legal guardians. Data was cleaned, verified, and entered into EpiData version 3.1 and analysis was done using SPSS version 24 statistical software.

**Result:**

Seven hundred and ninety-eight children agedd less than 18 years were involved with a response rate of 94.33 percent. More than one thirds (276, 34.59%) of children were displaced due to the conflict. Three hundred and thirty one (41.48%) children get diseased with the majority didn’t receive treatment. More than two thirds (557, 69.80%) of children had experienced violence of different types. One hundred and thirty four (41.23%) children had acute malnutrition with two third (66.42%) of them had severe acute malnutrition.

**Conclusion:**

Conflict had deadly impacts on the lives of children and adolescents. It causes massive displacement, lack of basic humanitarian needs, extreme level of violence, hunger and malnutrition, and lack of health services. The government and other national and international humanitarian aids should give special attention to children living in war zone of Amhara region. In addition, rehabilitation services and resilience training should be designed and provided to children affected by the conflict.

## Introduction

To date, conflict become a common phenomenon in the world affecting the lives of millions of children. Most of the conflicts are taking place in developing countries where 90% of the world’s children lives [1, 2]. Africa is the biggest conflict-affected region with the highest intensity in its Horn [3, 4]. In addition, many conflict cases were reported in Ethiopia mainly in most areas of Oromia region, Binishangulgumz, Ataye town for more than eleven cycles, and the Oromia special zone of Amhara region [5, 6]. Moreover, a very high-intensity armed conflict broke out in the northern part of the country in late 2021 which resulted in massive human rights violations and resource destruction. Furthermore, a large scale conflict is taking place in Amhara region which is continued to affect the lives of millions of children and adolescent [4, 7, 8].

Although conflict affects people of all ages and sexes, women and children are the most vulnerable groups of the society by the conflict [3, 9]. Evidences showed that more than two-thirds of the world’s children are living in conflict affected-regions [3]. Children living in conflict zone are suffering from the direct and indirect consequences of the conflict [8, 10]. They are suffering from extreme levels of violence, disabilities, hunger and malnutrition, denial of basic humanitarian needs, and lack of access to health services [11–13]. Studies has also showed that due to the continued conflict in the last decades two million children have been killed, four to five million disabled, twelve million were made homeless, more than one million became orphaned, and nearly ten million have been psychologically traumatized [8].

Evidences had revealed that conflict has severely impaired the social structure and social cohesion causing family and social disintegrations [14, 15]. Children with family and social disintegration lack social protections and they are at increased risk for neglect, abuse, violence, child labor, and trafficking [16–18]. In addition, children might watch the horror as their families and societies are fighting, fleeing, being wounded, or died in the conflict. Such overwhelming experiences will have then a damaging impact on their development, their attitude toward the society, their future relationship, and their outlook in life in general [19].

In 2019, over 45.7 million people were displaced worldwide due to the conflict. More than half of these conflict related displaced people were children aged less than 18 years. Nearly 42% of displaced children are found in Africa particularly in sub-Saharan African countries. Moreover, Ethiopia is ranking among the top three countries in child displacement [20–23]. Studies showed that displaced children face a number of problems including lack of access to the basic services, risk for violence, exploitation, abuse, and trafficking. They are also at higher risk of child labor, child marriage and family separation which all pose direct threats to their health and safety [24–26].

The healthcare delivery system remains a huge concern in conflict-affected regions of the world. This is due to the fact that conflict causes severe destruction of medical facilities, theft of essential medicines and supplies, blockage of transport and curfews all of which contributed to poor health care services in the conflict affected areas. In addition, conflict is associated with intense security issues which result in lack of healthcare personnel to provide health services [27–29]. As a result children in conflict areas remained untreated and are died from easily preventable disease [18, 30].

Food and nutritional insecurity is a major problem in conflict-affected areas [31, 32]. Studies showed that 75% of the global stunted and wasted children are found in conflict-affected regions [32–36]. In the past two decades the number of stunted children in conflict-affected countries of developing world has increased from 97.5 to 112.1 million [37, 38]. A significant number of child are continued to die from malnutrition worldwide in conflict ridden area. Malnutrition weakens the immune system and leaves children vulnerable to the killer diseases including cholera, pneumonia, and other infectious diseases. Furthermore, malnutrition causes impaired physical and cognitive development among children among survivors [39].

Children and adolescents living in conflict zone are suffering from extreme level of violence. In 2016, over 1 billion children aged < 18 years have experienced physical, sexual, or emotional violence due to conflict with the majority of which occur in the African countries [40]. Similarly, child violence including arbitrary killings, torture, forced marriage, abduction, rape, and trafficking are common experiences in conflict regions [41, 42]. Moreover, more than one-thirds of children who lived in war zone had experienced various forms of mental health problems insulting and threating with bad marks all of which then exposed children to depression, anxiety, posttraumatic stress disorders, and other behavioral related problems [43–45].

Conflict seems unavoidable phenomenon in Ethiopia and children are continued to suffer from this non-stoppable crisis. According to the report of the United Nation International Children’s Fund (UNICEF), more than 12.5 million children in Ethiopia are in need of humanitarian assistance. Admission to severe acute malnutrition has increased in the country, displacement has increased over time coupled with lack of basic services, poor shelter and sanitation. A significant number of child are suffering from the physical, psychological, and sexual violence in Ethiopia [46].

Although conflict has such deadly impacts on the lives of children and adolescent, there has been limited data regarding the situation of children and adolescent living in conflict affected regions. Consequently, no action has been implemented to protect the lives of children in conflict areas. Therefore, the finding from this study will provide an insight regarding the overall condition of children and adolescent in war zone of Amhara region. The finding could also provide baseline data to provide interventional measures to children affected by the conflict.

## Methods and materials

### Study setting and populations

A community-based cross-sectional study was conducted in conflict ridden areas of Amhara region from March to June 2022. Eight zonal administrations of the Amhara region were affected by the conflict and three of these conflict affected zones (North Wollo, South Wollo, and Dessie city administration) were selected by lottery method. Three Woredas were selected from North Wollo (Meket, Woldiya, and Gubalafto), 5 Woredas from South Wollo (Jama, Kalu, Albuko, Ambassel, and Dessie Zuriya), and 2 sub cities from Dessie city administration (Buanbua Wuha and Hote). Children aged less than 18 years who lived in conflict zones of Amhara region were included. Multi-stage random sampling methods were applied to recruit the study participants.

### Eligibility criteria

In this study children aged less than 18 years who lived in conflict affected zones of Amhara region during the conflict were included. However, children and adolescents who leave the area before the conflict for any reasons other than the conflict and retuned back to their usual residency after the conflict were excluded.

### Sample size determination

The sample size was determined by using a single population proportion formula by considering the assumptions Zα/2 = critical value for normal distribution at 95% confidence level which equals to 1.96 (z value at α = 0.05), estimated proportion (*p* = 50%), and absolute precision or margin of error 5% (d = 0.05).

$$\begin{array}{cc}no=\frac{{\left(\frac{Za}{2}\right)}^{2}*p\left(1-p\right)}{{d}^{2}};& \\ no=\frac{{\left(1.96\right)}^{2}*0.5\left(1-0.5\right)}{({0.05)}^{2}};& \underset{{\_}{\_}{\_}{\_}{\_}{\_}{\_}{\_}{\_}{\_}{\_}{\_}}{no=384}\end{array}$$

Considering 10% for the non-respondents the final sample size for the study was 423. Since we have used a multi-stage random sampling technique, we multiply the sample size by the design effect of 2 and the final sample size for the study was 846.

### Sampling technique

Eight zonal administrations of the Amhara region were affected by the conflict. Three of these conflict-affected zones (North Wollo, South Wollo, and Dessie City Administration) were selected by lottery method. Three Woredas were selected from North Wollo namely Meket, Woldiya, and Gubalafto. Five Woredas from South Wollo namely Jama, Kalu, Albuko, Ambassel, and Dessie Zuriya. Two Sub-cities were selected from Dessie city administration namely Buanbua Wuha and Hote sub cities. From each selected Woredas and sub cities Kebeles were selected randomly. Finally, households having children aged less than 18 years were selected by systematic random sampling technique from each selected Kebeles. However, in the sampled households with more than one child aged less than 18 years only one child was included by lottery method.

### Data collection tool and procedure

The data collection tool was adopted from studies conducted in conflict zones of the world which was prepared for similar purposes [47]. The tool was tested and modified accordingly to make it suitable to the study objectives. Assent was filed by the legal guardians of the child after a detailed explanation of the purpose, risk, and benefits of the study. Participants were informed that participation is voluntary and they can withdraw at any time with no consequences. Participants were also informed that they can choose not to answer any questions they don’t want to answer. The study participants are informed that data will be kept anonymous and no one has access to the data without their voluntary consent.

### Data analysis

Data were verified, coded, and entered to EpiData Software version 3.1 and was exported to SPSS version 24 Software for analysis. Data transformation, editing and cleaning were done before the analysis. Finally, descriptive statistics like frequency and percentages were computed and presented through narrations, tables, and graphs.

### Operational definition

#### Psychological violence

Children experiencing any of the following behaviors directed towards them: shouted at, told they are not loved, locked in, insulted or called bad names, made fun of in front of others, ignored for prolonged periods of time.

#### Physical violence

Children experiencing any of the following: being slapped or having things thrown at them; being pushed, cornered, or having their ears or hair pulled; and being smacked or slapped, being hit with a fist or object; being kicked, dragged or given a beating; being burned (or attempted burning); or threatened with a pistol, knife or other weapons.

#### Sexual violence

Children experiencing any of the following touched sexually caressed some part of the body or forced to touch them sexually, or being raped, sexual slavery, forced prostitution, forced pregnancy, or experiencing sexual torture. Children were considered to have experienced sexual violence if they were at least five years younger than the abuser and if the abuser was at least 12 years of aged and this was assured by history.

#### Malnutrition

Children diagnosed for one of the following: MUAC < 12.5 cm or WFH < -2z-score or the presence of bilateral pitting edema.

#### Displacement

The number of children who leave their usual residential areas due to the conflict.

#### Illness/disease

The number of children or adolescents who were sick during the time of conflict.

#### Treatment

Any form of health services provided to children and adolescent during the conflict.

## Result

### Sociodemographic features of children

Seven hundred and ninety-eight children and adolescents were involved with a response rate of 94.33 percent. Four hundred and eight (51.13%) children were aged ≤ 8 years and 390 (48.87%) were children aged 8–17 years. The mean age of the participant was 8.45 years (SD: ± 5 years. More than half of the participants (57.64%) were male and nearly two thirds (64.42%) were urban dwellers.

### Displacement and associated problems

The study showed that 276 (34.59%) children were displaced due to the conflict. Nearly half (48.91%) of the displaced children had experienced other health and health related problems including lack of basic humanitarian needs (58.70%), lack of health services (24.64%), and illness and trauma (13.04%). The study revealed that 105 (38.04%) children face multiple health-related problems while they are displaced.

### Disease profile of children in conflict zones

The health status of children was assessed whether they have experienced any form of illness during the conflict using yes or no questions. Consequently, 331(41.48%) children and adolescents had experienced illness or disease during the conflict. Of these, 124 (37.46%) children had abdominal pain or Diarrhea, 79 (23.87%) had respiratory problem, 44 (13.29%) febrile illness, 15 (4.35%) ear or eye problems, 38 (11.48%) had depressions, and the rest 31 (9.37%) had other health problems including trauma, skin problems, allergy.

Three hundred and twenty five children aged6-59 months were screened for malnutrition using mid-upper arm circumference (MUAC), weight for height (WFH), and bilateral pitting edema. The finding showed that 134 (41.23%) children had acute malnutrition (wasting) and more than one-thirds (66.42%) of them had severe acute malnutrition. The finding had also revealed that majority of children (78.36%) with acute malnutrition didn’t receive treatment for their illness.

### Physical violence

Physical violence was assessed using 14 trauma questions with yes or no responses and ‘yes’ response to any of the trauma questions were reported as having physical violence. Consequently, more than half children living in conflict zone (*n* = 423, 53.01%) had experienced physical violence. Majority of them (*n* = 362, 85.58%) had experienced multiple traumatic events. The most frequently reported physical traumas were pushed or /kicked (43.86%), hit or beat (37.22%), and made kneel for punishment (29.20%) (Table 1).

**Table 1:** Physical trauma among children and adolescent aged < 18 years in war zones of Amhara region February 2022

Physical violence	Yes, n (%)	No, n (%)
Pushed, grabbed or kicked	350 (43.86%)	448(56.14)
Hit, beat or spanked	297 (37.22)	501 (62.78)
Choked or tried to down	77 (9.65)	721 (90.35)
Burned or scaled	20 (2.51)	778(97.49)
Locked, tied or chained up	50 (6.27)	748 (93.73)
Pulled hair, pinched or twisted ear	158 (19.80)	640 (80.20)
Forced to hold a heavy load	176 (22.06)	622 (77.94)
Threw an object to you	151 (18.92)	647 (81.08)
Hit with a closed fist	144 (18.05)	654 (81.95)
Crushed fingers or hands	183 (22.93)	615 (77.07)
Made stand/kneel for punishment	225 (29.20)	573 (71.80)
Made stay in hot or cold place	55 (6.89)	743 (93.11)
Took away of your food/drink	61 (7.64)	737 (92.36)
Tried to cut you with sharp object	122 (15.29)	676 (84.71)

### Psychological violence

Psychological violence was assessed using 12 trauma questions with yes or no responses and ‘yes’ response to any of these 12 questions was reported as having psychological violence. Thus, the finding revealed that nearly two thirds (*n* = 535, 67.04%) of children had experienced psychological violence. The majority of them (*n* = 492, 91.96%) had experienced multiple psychological violence. The most frequently reported psychological violence was shouting at them (61.53%), insulting (55.89%), calling with rude names (43.36%), and threatening with bad marks (41.23%) (Table 2).

**Table 2:** Psychological trauma among children and adolescent aged < 18 years in war zones of Amhara region February 2022

Psychological violence	Ye, n (%)	No, n (%)
Shouted/screamed	491 (61.53)	307 (38.47)
Threatened with bad marks	329 (41.23)	469 (58.77)
Called in rude hurtful names	346 (43.36)	452 (56.64)
Insulted	446 (55.89)	532 (41.11)
Stole or broke belongings	299 (37.47)	499 (62.53)
Isolated from family	96 (12.03)	702 (87.97)
Hurtful prejudice (gender, ethnicity etc.)	109 (13.66)	689 (86.34)
Made ashamed/embarrassed	260 (32.58)	538 (67.42)
Made ashamed/embarrassed in front of others	247 (30.95)	55 (69.05)
Threatened to kill or hurt	196 (24.56)	602 (75.44)
Kidnapped	142 (17.79)	656 (82.21)
Witnessed while a person is being harmed/killed	212 (26.57)	586 (73.43)

### Sexual violence

A total of 11 questions with binary responses (yes or no) were used to assess sexual violence. The finding revealed that 96 (12.03%) children had experienced sexual violence. More than two-thirds of these children 66 (68.75%) had experienced multiple incidences of sexual violence. The most frequently reported sexual violence were touched or caressed sexually (*n* = 34, 35.42%), tried to have sex with them (*n* = 29, 30.21%), and being raped (*n* = 14, 14.58%).

### Child Health services during the conflict

The health services for children during the conflict were assessed by asking whether the child had received health services during the conflict. Subsequently, 268 (80.97%) children didn’t receive any form of treatment for their illness. The major reasons for not receiving treatment were due to closed or damaged health services (68.40%), restricted movement or fear of the being injured or killed (24.54%), and due to being displaced and no services available in the refugee camp (7.06%).

## Discussion

This study showed that children face a number of problems in conflict zones of Amhara region. The finding is supported by studies in different conflict-affected regions of the world where children are suffering from the direct and indirect consequence of conflict including displacement, lack of basic humanitarian needs, violence of different types, as well as hunger and malnutrition [11–13]. The reason could be children are not yet mentally, physically, and emotionally developed as a result they are unable to cope the negative consequences of the conflict and are more likely to experience risks. This suggested that special attention should be given to children who are living in conflict regions of Ethiopia and in the world at large.

More than one-third of children (34.59%) were displaced due to the conflict. The finding is similar to studies in most-conflict affected settings of the world [8, 9]. The reason could be families or child legal guardians might use displacement as a protection mechanism for children form the harmful impacts of the conflict. However, in real situation, children face a number of problems while displacing including lack of basic humanitarian need, exposure to disease and lack of access to medical service, and hunger and malnutrition. The finding implied that majority of the displaced people during the conflict are children and hence national and international organizations should take attention to these majority displaced children.

This study showed that more than one-thirds of children (41.48%) get diseased during the conflict and more than three-fourth (80.97%) didn’t receive treatment for their illness. The finding is supported by the study in conflict-affected regions of the world [27–29]. The reason could be due to the destruction of the health care institutions or theft of drugs and supplies or it might be associated with lack healthcare personnel due to security problems to provide treatment and care for children. The reason could also be due to the restriction of movement due to the fear of being injured or killed or it might be due to lack of money to pay for the health care services. The finding pointed out that child protection strategies should be designed and implemented to save the lives of millions of children and adolescent in conflict ridden area of the world.

More than two-thirds (69.80%) of children and adolescents had experienced violence. The finding is supported by a systematic review of the global prevalence where violence is a common occurrence among children in conflict zones of the world [40]. The reason could be children and adolescent are not yet physically, mentally, and emotionally competent to protect themselves from the detrimental events of the conflict. As a result, child protection measures should be designed and implemented to protect the lives of children and adolescents in conflict zones.

Nearly two third (67.04%) of children had experienced psychological trauma. The finding is similar to a report of the world health organization [19], a study in the war zone of the world [45], a study in Colombia [15], a study in Palestine [43], and another study in conflict zones of the world [48]. This is the fact that children who lived in the war zone are exposed to the terrible effect of war, witness injured or dead people, observe destruction of institutions, and other shocking events of the conflict. Consequently, children involved in or witnessed such disturbing events are more likely to experience psychological trauma. The higher estimate of psychological violence in children could be attributed to their mental and emotional under development. Consequently, rehabilitation service and resilience training should be given to children living in conflict region [9].

Although it was difficult to draw accurate data and there might be under reports on sexual-related violence, the finding revealed that one in eight children had experienced sexual violence. The finding is supported by the gender-based studies in post-conflict areas of the world [41, 42]. The reason could be the armed forces might use sexual violence as a means of weapon and ethnic cleansing. Additionally, young women are biologically susceptible to sexual violence which put them at a higher risk for sexual violence than other groups of children.

### Limitation of the study

The cross-sectional study is also vulnerable to recall bias and dishonesty which might lower the actual figure of violence in the study area. In addition, children and adolescents might hide their violent acts due to the fear of rejection by society. This could also lower the actual figure of child and adolescent violence in the study area.

## Conclusion

Conflict causes enormous health and health related impacts on children and adolescents. It causes displacement, lack of basic humanitarian needs, lack of health services, violence of different type including the physical, psychological, and sexual violence, hunger and malnutrition. The government and other national and international humanitarian aids should give due attention to children who lived in conflict regions.

## Data Availability

All relevant data is included in the manuscript.

## Abbreviations

MUAC: Mid Upper Arm Circumference
WFH: Weight for Height
